# Paradoxical pustular psoriasis following dupilumab therapy for bullous pemphigoid: A case report

**DOI:** 10.1016/j.jdcr.2026.04.005

**Published:** 2026-04-14

**Authors:** Melanie D. Medina-Figueroa, Iancarlos Jiménez-Sacarello, Mariana Sadurní-García, Rafael Martín-García, Marely Santiago-Vázquez

**Affiliations:** a Department of Dermatology, University of Puerto Rico School of Medicine, San Juan, Puerto Rico; b School of Medicine, University of Puerto Rico, Medical Sciences Campus, San Juan, Puerto Rico

**Keywords:** bullous pemphigoid, dupilumab, pustular psoriasis

## Introduction

Dupilumab is a monoclonal antibody that inhibits type 2 helper T-cell inflammation by targeting the interleukin 4 receptor alpha (IL-4Rα) subunit, thereby blocking IL-4 and IL-13 signaling. It was first approved for moderate-to-severe atopic dermatitis (AD) and has since gained expanded indications in dermatology, most recently for bullous pemphigoid (BP). As its use has increased worldwide, dupilumab has proven to be an effective and generally well-tolerated therapy. However, an increasing number of reports have described dupilumab-associated psoriasis and psoriasiform eruptions in patients treated for atopic dermatitis.^1^^,^^2^ One of these manifestations is pustular psoriasis, characterized by superficial sterile pustules on an erythematous base, occurring with or without concomitant psoriasis vulgaris. We report a rare case of a dupilumab-induced paradoxical pustular flare in a patient with pre-existing psoriasis treated for bullous pemphigoid.

## Case report

A 64-year-old Hispanic woman with a history of hypertension and longstanding plaque psoriasis was evaluated by our service after presenting to the emergency department with a 2-week history of a progressive erythematous eruption associated with tense bullae involving the trunk, back, and upper and lower extremities.

A punch biopsy demonstrated a subepidermal vesicle with a mixed inflammatory infiltrate rich in eosinophils. Direct immunofluorescence revealed linear IgG and C3 deposition along the dermoepidermal junction. Serologic testing showed elevated BP180 antibody levels (113 U) and BP230 antibody levels (26 U). These clinical, histopathologic, and immunologic findings supported a diagnosis of BP.

She was initiated on prednisone 40 mg daily and triamcinolone acetonide 0.1% cream. Although gradual tapering of prednisone was attempted, disease flares recurred upon discontinuation. Given the relapsing course and the need for a steroid-sparing strategy, dupilumab therapy was initiated with a 600 mg loading dose followed by 300 mg every 2 weeks. Table I summarizes the treatment course. Of note, at the time of dupilumab initiation, her plaque psoriasis had been well controlled for several years with emollients alone, with no active lesions and a Physician Global Assessment score of 0. Prednisone 10 mg daily continued during dupilumab initiation given persistent BP flare ups.

**Table I:** Timeline of patient’s disease and treatment course

Timepoint	Clinical course
February 2025	Evaluated by our Dermatology service in the ER for a diffuse erythematous rash with associated vesicles and bullae on the upper extremities and trunk. Punch biopsies were performed, and the patient was started on prednisone 40 mg daily.
March 2025	The patient reported new lesions. Biopsy results were positive for bullous pemphigoid. Prednisone increased from 40 mg daily to 50 mg daily.
April 2025	No new lesions were reported. Prednisone decreased from 50 mg daily to 40 mg daily for 2 wk, then 30 mg daily for 2 wk.
May 2025	No new lesions were reported. Prednisone decreased from 30 mg daily to 10 mg daily for 2 wk, then 10 mg every other d.
June 2025	No active lesions. Prednisone was discontinued, and the patient was started on minocycline 100 mg daily for 4 wk.
July 2025	Patient reported new lesions after discontinuing prednisone. Dupixent was prescribed: 600 mg on d 0, then 300 mg every 2 wk. Minocycline was discontinued, and prednisone 10 mg daily was restarted while awaiting Dupixent approval.
July 20, 2025	Initial dose of Dupixent administered.
August 2025	Patient presented with new-onset lesions on the bilateral lower extremities consisting of multiple coalescing, well-defined erythematous plaques with overlying scaling and pustules. A punch biopsy was performed. Patient advised to hold Dupixent. Prednisone 10 mg daily and minocycline 100 mg daily were started.
September 2025	Biopsy confirmed pustular psoriasis. Ixekizumab started: 160 mg once, then 80 mg every 2 wk. Prednisone tapered from 10 mg daily to 5 mg daily. Minocycline discontinued. Dupixent remained held.

After the third dose of dupilumab, there was marked improvement in her bullous pemphigoid lesions. However, approximately 2 weeks after starting dupilumab, she developed a new pustular eruption localized to the lower extremities. She denied fever, arthralgias, malaise, gastrointestinal symptoms, or other systemic complaints. A complete blood count obtained at that time was within normal limits.

**Fig 1:**
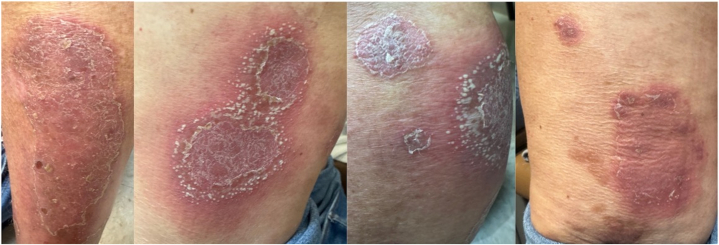
Multiple erythematous plaques with overlying silvery scale surrounded by sterile pustules on the lower extremities.

On examination, there were erythematous annular patches involving the upper extremities. The lower extremities demonstrated multiple erythematous thin plaques, several with annular morphology and scattered pustules (Fig 1), in addition to erythematous, thickly scaly plaques on the bilateral plantar surfaces. There was no evidence of nail, mucosal, or articular involvement.

**Fig 2:**
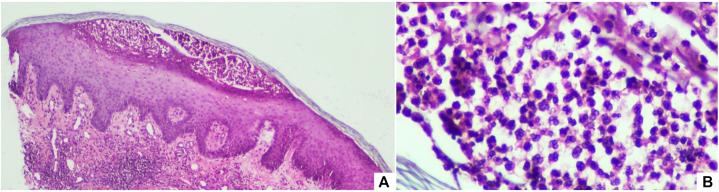
**A,** Psoriasiform epidermal hyperplasia with parakeratosis and spongiosis, small vesiculopustules, and lymphocytic dermal infiltrate (H&E, 10×). **B,** Intracorneal neutrophilic collection (H&E, 40×).

A punch biopsy obtained from a lesion on the lower extremity revealed psoriasiform epidermal hyperplasia with parakeratosis and spongiosis, accompanied by small vesiculopustules and a superficial lymphocytic dermal infiltrate. Intracorneal neutrophilic aggregates were also identified (Fig 2). These findings were consistent with pustular psoriasis.

**Fig 3:**
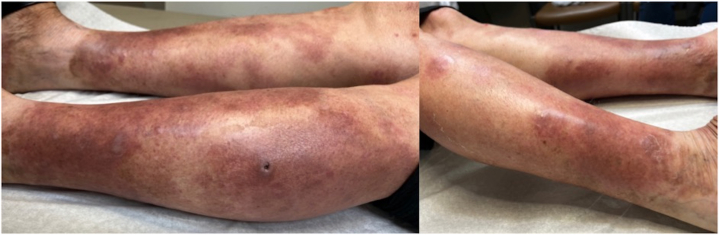
Improvement of psoriatic lesions 2 weeks after initiation of ixekizumab treatment.

Given extensive pustular psoriasis lesions and limited BP lesions, dupilumab was subsequently discontinued, and therapy with ixekizumab was initiated at a loading of 160 mg, followed by 80 mg administered every 2 weeks. Prednisone gradually tapered to 5 mg daily during this transition to prevent BP flare ups.

Within 2 weeks of starting ixekizumab, the patient demonstrated significant clinical improvement, with complete resolution of the pustular lesions (Fig 3). At 5 months of follow-up, she remains on ixekizumab with sustained remission and no recurrence of either psoriatic or bullous pemphigoid lesions.

## Discussion

Paradoxical psoriasiform eruptions have been increasingly reported in patients treated with dupilumab. Although initially approved for atopic dermatitis, dupilumab was recently approved for the treatment of BP. Interestingly, a subset of patients treated with dupilumab for AD develop de novo psoriasis or exacerbations of pre-existing disease, including pustular phenotypes.^2^ Recent studies indicate that Th2 blockade by dupilumab may cause a Th1/Th2 imbalance, leading to an unopposed Th1/Th17 response, resulting in increased IL-17/IL-23 signaling and neutrophil dominant inflammation pathways central to psoriasis.^1^ Patients with prior psoriasis appear especially susceptible due to pre-existing immune priming of this pathway.^3^

The coexistence of BP and psoriasis has been well described and highlights overlapping immune dysregulation. Epidemiologic studies show a three-fold increased risk of BP in patients with psoriasis.^4^ Psoriasis typically precedes BP. Pustular psoriasis occurring with BP is uncommon, and reported triggers include PUVA therapy, pembrolizumab, and withdrawal of systemic corticosteroids.^5^ Proposed mechanisms for concomitant BP and psoriasis include enzymatic degradation of basement membrane proteins in psoriatic plaques, which may expose BP antigens, as well as increased IL-17 and IL-36 expression within BP lesions.^6^ These findings have led to successful use of IL-17 and IL-36 targeted therapies in selected patients.^7^

Management of concurrent psoriasis and BP varies depending on severity and phenotype. IL-17 inhibitors such as secukinumab and ixekizumab have achieved remission of both disorders in several reports.^8^ Janus kinase inhibitors including baricitinib, upadacitinib, and tofacitinib have also been effective in isolated cases.^9^ Dupilumab use has also been reported combination with systemic corticosteroids, methotrexate, topical corticosteroids, and sequential IL-36 inhibition. For pustular psoriasis combined with BP, IL-36 blockade with spesolimab produced rapid improvement in 2 published cases.^5^^,^^7^

Reports of dupilumab-induced psoriasis in patients treated for BP are rare. To our knowledge, only 3 patients have been described to date (Table II).^9^^,^^10^ No previous cases of a dupilumab induced pustular psoriasis in this setting have been described. The leading hypothesis parallels findings in atopic dermatitis: dupilumab may shift the immune response from a type 2 dominant profile toward a Th1 and Th17 pattern with increased IL-23 and IL-36 activity. Because experience remains limited, there are no standardized management recommendations. One case reported successful use of upadacitinib therapy with resolution of psoriatic and BP lesions.^9^ In our case, discontinuation of dupilumab and initiation of ixekizumab resulted in complete clearance of pustules after 2 weeks and sustained remission of both psoriasis and BP.

**Table II:** Reported cases of dupilumab induced psoriasis during bullous pemphigoid treatment

	Type of study	# of cases	Age/Sex	Ethnicity	Psoriasis variant	Prior hx of psoriasis?	Onset after dupilumab initiation	Treatment
Zhao L, et al (2023)^10^	Retrospective cohort	2	N/A	N/A	N/A	N/A	N/A	N/A
Su F, et al (2024)^9^	Case report	1	66/M	N/A	N/A	Yes	2 wk	Upadacitinib
Our case	Case report	1	64/F	Hispanic	Pustular	Yes	2 wk	Ixekizumab

Although the patient had pre-existing plaque psoriasis and was undergoing a prednisone taper, several features suggest dupilumab as the trigger for her paradoxical pustular eruption. The lesions appeared 2 weeks after dupilumab initiation and resolved rapidly after its discontinuation and initiation of ixekizumab, consistent with a dechallenge effect. She had previously undergone corticosteroid tapering without the development of pustular lesions, making steroid reduction alone an unlikely explanation. No new medications or infections were identified, and her psoriasis had been stable prior to our evaluation. While we acknowledge the limitations of a single case report, including underlying dermatologic conditions and concomitant therapies, the temporal relationship, clinical course, and exclusion of other triggers support dupilumab as the precipitating factor of this paradoxical psoriatic exacerbation.

As dupilumab continues to gain clinical indications, it is essential to recognize its potential to induce paradoxical psoriatic reactions. To our knowledge, this is a unique case of dupilumab-induced pustular psoriasis in a patient treated for BP. Clinicians should exercise caution in patients with a history of psoriasis and maintain a high index of suspicion when new inflammatory lesions appear shortly after starting dupilumab. Further mechanistic studies are needed to clarify how IL-4Rα blockade alters immune balance and predisposes certain individuals to Th1 and Th17 driven inflammation.

## Conflicts of interest

None disclosed.
